# Clinical features and pregnancy outcomes in women with aortopulmonary window defect: case series

**DOI:** 10.1093/ehjcr/ytad501

**Published:** 2023-10-09

**Authors:** Giunai Gahraman Sefiyeva, Diana Sergeevna Malishevskaya, Aigul Nasiridinovna Chynybekova, Andrey Evgenievich Bautin, Ulyana Mikhailovna Shadrina, Daria Vladimirovna Alekseeva, Ekaterina Leonidovna Urumova, Olga Borisovna Irtyuga

**Affiliations:** Almazov National Medical Research Centre, 2 Akkuratova Street, Saint Petersburg 197341, Russia; Almazov National Medical Research Centre, 2 Akkuratova Street, Saint Petersburg 197341, Russia; Almazov National Medical Research Centre, 2 Akkuratova Street, Saint Petersburg 197341, Russia; Almazov National Medical Research Centre, 2 Akkuratova Street, Saint Petersburg 197341, Russia; Almazov National Medical Research Centre, 2 Akkuratova Street, Saint Petersburg 197341, Russia; Almazov National Medical Research Centre, 2 Akkuratova Street, Saint Petersburg 197341, Russia; Almazov National Medical Research Centre, 2 Akkuratova Street, Saint Petersburg 197341, Russia; Almazov National Medical Research Centre, 2 Akkuratova Street, Saint Petersburg 197341, Russia

**Keywords:** Pregnancy, Aortopulmonary window, Eisenmenger syndrome, Case report

## Abstract

**Background:**

Aortopulmonary window is a rare congenital heart defect that results in severe pulmonary arterial hypertension (PAH), Eisenmenger syndrome, and congestive heart failure in the first months of life. Pregnancy is absolutely contraindicated in the patients with this condition.

**Case summary:**

This paper describes two clinical cases of pregnancy in patients (28 and 20 years old) with aortopulmonary window defect, severe PAH, and Eisenmenger syndrome that ended in preterm delivery by caesarean section. One patient died in the postpartum period due to progression of right ventricular heart failure. The younger patient survived childbirth and the postpartum period; later, she continued therapy at the PAH centre.

**Discussion:**

We describe unusual cases of clinical features in pregnant women with aortopulmonary window defect. Due to the rare occurrence and low survival rate of patients with uncorrected aortopulmonary window defect, descriptions of clinical cases of this defect in adults are very rare. It is very important to note the necessity of observation of these patients in specialized centres by a multidisciplinary team of healthcare professionals, due to the high risk of cardiovascular, obstetric complications, and death.

**Learning points**

- The mortality rate of patients with aortopulmonary window defect in the structure of congenital heart defects without surgical repair in the first year of life reaches 40%.
- According to current clinical guidelines on the management of pregnant patients with cardiovascular diseases, the World Health Organization classifies pulmonary arterial hypertension as class IV risk and pregnancy is absolutely contraindicated.
- If the patients categorically refuse to terminate the pregnancy, they should be observed in specialized centres by a multidisciplinary team of healthcare professionals.

## Introduction

The proportion of aortopulmonary window defect in the structure of congenital heart defects comprises 0.1–0.2%^1,2^; the mortality rate of patients without surgical repair in the first year of life reaches 40%.^3,4^ According to current clinical guidelines on the management of pregnant patients with cardiovascular diseases, the World Health Organization classifies pulmonary arterial hypertension (PAH) as class IV risk (40–100% probability of cardiovascular events in the mother). Pregnancy is absolutely contraindicated in the patients with this condition.^5^

## Summary figure

**Figure:**
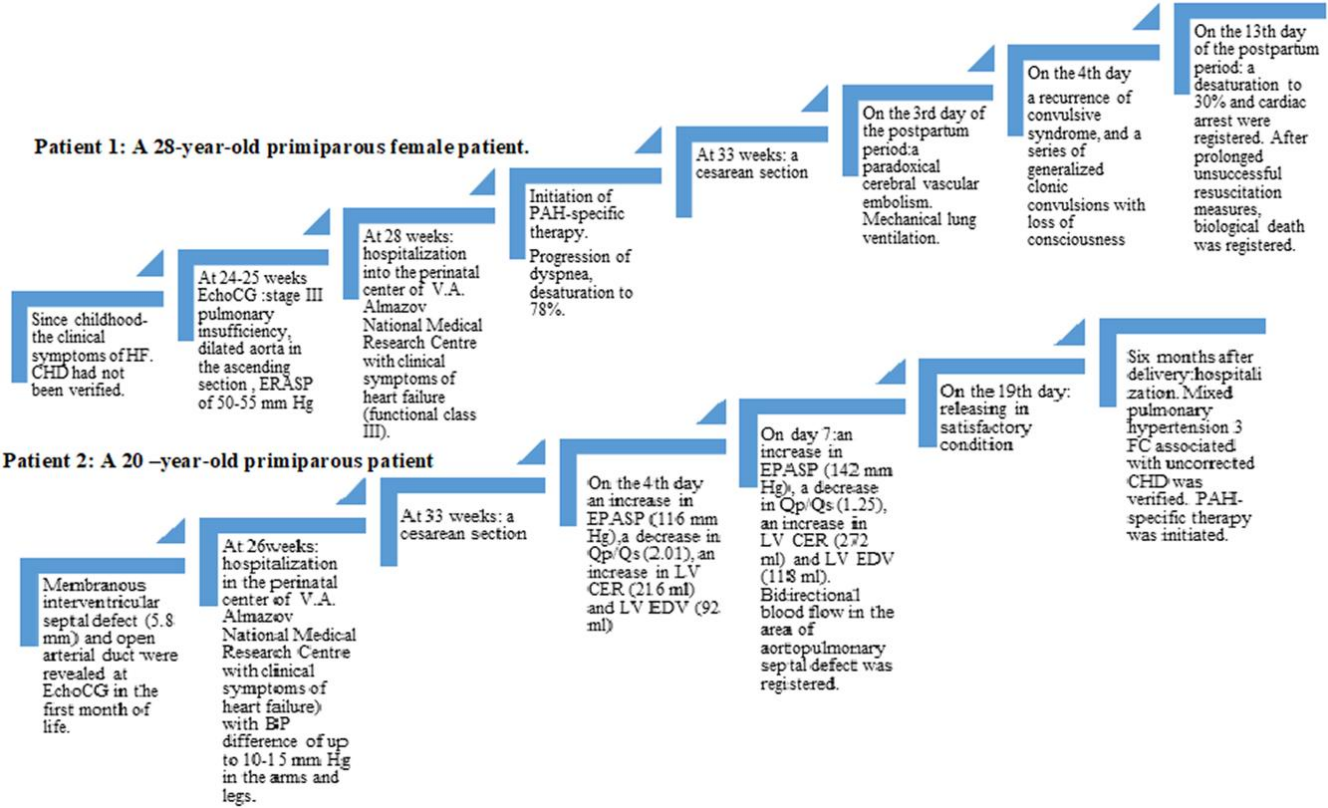


## Materials and methods

The paper reviews two clinical cases of pregnant patients (28 and 20 years old) with aortopulmonary window defect with Eisenmenger syndrome. Congenital heart disease (CHD) was verified by imaging techniques: transthoracic and transoesophageal echocardiography (EchoCG), non-contrast magnetic resonance imaging (MRI) of the heart and aorta and, after delivery, computed tomography (CT) of the aorta with contrast. The babies were delivered by caesarean section in the expert centre for pregnancy and cardiac disease. One of the patients underwent catheterization of the right heart chambers after delivery. The patients’ clinical and laboratory data were regularly monitored; electrocardiography (ECG), EchoCG, Dopplerometry, and fetometry were conducted.

## Results

### Patient 1

A 28-year-old primiparous female patient at 28–29 weeks gestation was hospitalized into the perinatal centre of V.A. Almazov National Medical Research Centre with clinical symptoms of heart failure [FC (functional class) III]. The symptoms had been observed since childhood. Congenital heart disease had not been verified by previous examinations.

Present pregnancy is at the age of 27; EchoCG (24–25 weeks) showed stage III pulmonary insufficiency, dilated aorta in the ascending section (up to 50 mm), estimated pulmonary artery systolic pressure (ERASP) of 50–55 mm Hg, and left ventricular ejection fraction (LVEF) 62%. Due to the revealed changes, the patient was transferred to the expert centre for pregnancy and cardiac disease.

Examination showed satisfactory general state and body mass index of 19 kg/m^2^. Acrocyanosis at rest was found. Vesicular breathing was respiratory rate (RR) = 20/min and SpO_2_ at rest was 87%. Pulse was rhythmic with heart rate 82 b.p.m, and blood pressure (BP) was 100/60 mmHg. Auscultation showed a systolic murmur on the left sternal border. The abdomen was enlarged according to the gestational age. Foetal ultrasound and Dopplerometry (29 weeks) showed symmetrical foetal growth retardation. As the pregnancy progressed, there were signs of preeclampsia, reduced exercise tolerance, and desaturation to 85%. Pulmonary arterial hypertension–specific therapy with sildenafil was initiated. There was a progression of dyspnoea and desaturation to 78%. At 33 weeks of gestation, a caesarean section was performed. The patient’s intraoperative course was without complications.

Activation of the patient on the third day of the postpartum period led to a generalized convulsive seizure accompanied by loss of consciousness, desaturation to SpO_2_ = 40%, arterial hypertension, and tachycardia. The episode was treated as a paradoxical cerebral vascular embolism. The patient was intubated and transferred to mechanical lung ventilation (MLV). Sedative and anticoagulant therapy was administered, and PAH-specific therapy with inhaled iloprost was intensified. On the same day (the third postpartum day), the condition stabilized and the patient was extubated. On the fourth day, there was a recurrence of convulsive syndrome and a series of generalized clonic convulsions with loss of consciousness; there was desaturation to 60% on the sixth day. The patient was transferred to MLV with continuous infusion of barbiturates. In the course of ongoing MLV, the dynamics showed SpO_2_ decrease to 64%, increasing right ventricular heart failure; inotropic therapy with dobutamine was initiated. Subsequently, repeated episodes of increasing right ventricular heart failure at minimum exertion with desaturation to 45% were registered. FiO_2_ was increased to 100%, as well as the inotropic support.

**Figure 1:**
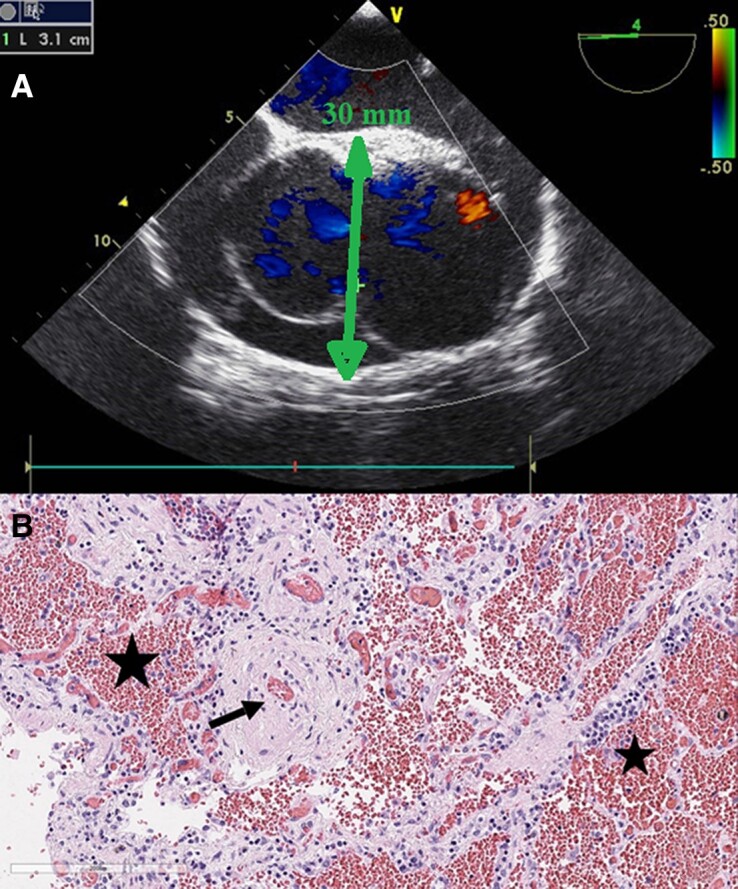
**Patient 1.** (*A*) Transoesophageal echocardiography at 29 5/7 weeks: ‘Supravalvular location of aortopulmonary junction up to 30 mm with balanced blood flow (truncus arteriosus type)’. (*B*) Concentric narrowing of the small-calibre pulmonary artery lumen due to wall fibrosis (arrow), intraalveolar haemorrhage as a demonstration of disseminated intravascular coagulation syndrome (asterisks), ×200 increase.

On the 13th day of the postpartum period, a desaturation to 30% and cardiac arrest were registered. After prolonged unsuccessful resuscitation measures, biological death was registered. A complex congenital heart defect with a large defect of the aortopulmonary septum was identified as the main cause of death with cardiopulmonary failure, disseminated intravascular coagulation (DIC) syndrome being the immediate cause of death (*Figure 1B*).

### Patient 2

A 20-year-old primiparous patient was hospitalized in our centre at 26 weeks of gestation with clinical symptoms of heart failure, class II by the New York Heart Association (NYHA) with blood pressure difference of up to 10–15 mm Hg in the arms and legs. Membranous interventricular septal defect (5.8 mm) and open arterial duct were revealed at EchoCG in the first month of life.

The patient categorically refused to terminate the pregnancy; she was referred to the expert centre for pregnancy and cardiac disease. Examination showed satisfactory general condition. Desaturation was not determined and foetal Dopplerometry was normal. Laboratory test results were normal, and NT-proBNP = 118.9 pg/mL. Echocardiography (26 weeks): significant left heart dilatation. Left atrium (LA), 49 mm; LA volume index, 56 mL/m^2^. Left ventricle: interventricular septum, 8–15 mm; PW, 8 mm; EDD, 59 mm; ESD, 42 mm, EDV, 261 mL, and ESV, 105 mL. The right chambers were not enlarged. The right ventricle (RV) was 10 mm, and LVEF was 60%; tricuspid annular plane systolic excursion (TAPSE) was 2.6 cm. In the ascending section, the aorta formed a single vessel with the pulmonary artery; vessel diameter was up to 57 mm (aortopulmonary junction). There was grade 2 aortic insufficiency. Estimated pulmonary artery systolic pressure (EPASP) was 128 mm Hg.

**Figure 2:**
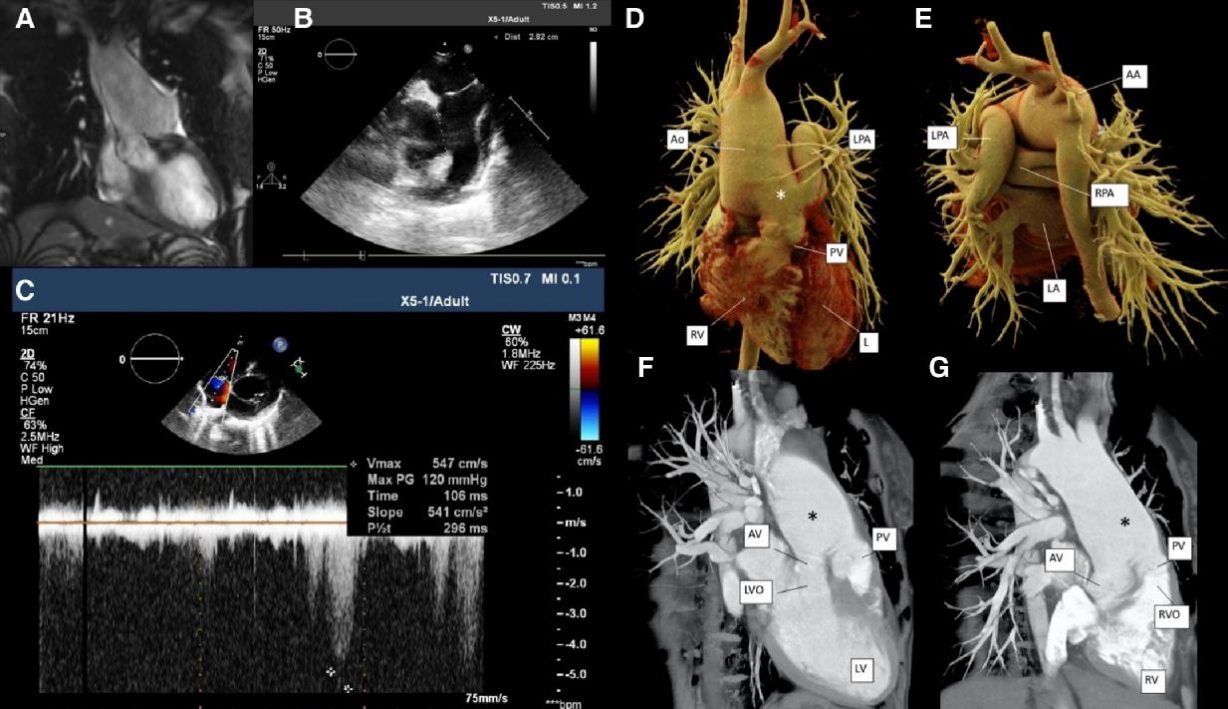
**Patient 2.** (*A*) Non-contrast magnetic resonance imaging (MRI) of the aorta at 32 3/7 weeks: ‘The ascending aorta and pulmonary trunk are communicating for 35 mm after 5–10 mm of divergence’. (*B*, *C*) Echocardiography 6 months after delivery: ‘Defect between aorta and pulmonary artery up to 41 mm with predominantly right-to-left shunt. Qp/Qs of 0.8’. (*D*, *E*) Electrocardiogram (EСG)-gated computed tomography angiography cinematic rendering (16 day postpartum). RV, right ventricle; LV, left ventricle; Ao, aorta; PV, pulmonary valve; LPA, left pulmonary artery. Star, aortopulmonary fenestration. Electrocardiogram-gated computed tomography angiography was performed and revealed two atriums and two ventricles with concordant connection. The aorta took its origin from the left ventricle and the pulmonary artery from the right ventricle. The location of the aortic and of the pulmonary valves was typical. From the level of the aortic sino-tubular junction, the large aortopulmonary fenestration with length of about 48 mm was found. Above, the fenestration aorta was enlarged to 60 mm. And the pulmonary artery was divided to the right and left branches directly after the fenestration. (*F*, *G*) Electrocardiogram-gated computed tomography angiography volume rendering technique (VRT) soft tissue reconstruction (16 day postpartum). LV, left ventricle; LVO, left ventricular outflow tract; RV, right ventricle; RVO, right ventricular outflow tract; AV, aortic valve; PV, pulmonary valve. Star, aortopulmonary fenestration. There was an extensive defect of the aortopulmonary septum of 4.6 × 4.8 cm from the level of the sino-tubular zone and significant dilation of the ascending thoracic aorta (above the defect level, the diameter was 6.4 × 6.1 cm).

Since no negative dynamics were registered by clinical, laboratory, or instrumental tests until 30 weeks of gestation, the pregnancy was prolonged up to 32 weeks. Data on EchoCG are shown at *Figure 2*.

After 33 weeks of gestation, there were appeared episodes of shortness of breath in the supine position and the episodes of dizziness with minimal physical exertion. At 33 weeks of pregnancy, caesarean section was performed.

Vasopressor and inotropic support was initiated on Day 1 of the postpartum period. On Days 4 and 7, there was an increase in estimated pulmonary artery systolic pressure (116 and 142 mm Hg, respectively), a decrease in Qp/Qs (2.01 and 1.25, respectively), and an increase in LV CER (216 and 272 mL, respectively) and LV EDV (92 and 118 mL, respectively). Bidirectional blood flow in the area of aortopulmonary window defect was registered. The patient’s condition was satisfactory. Data on CT angiography of the aorta are shown in *Figure 2D–G*. The patient categorically refused to have right heart catheterization (RHC). She was released in satisfactory condition on the 19th day of postpartum period without drug therapy and with the recommendation to have a planned RHC.

Six months after delivery, the patient was re-hospitalized. NT-proBNP was 472.1 pg/mL. Data on EchoCG are shown in *Figure 2B* and *C*. Thus, mixed pulmonary hypertension 3 FC associated with uncorrected congenital heart disease was verified. Pulmonary arterial hypertension–specific therapy was initiated of sildenafil 60 mg/day. Nine months after delivery, the patient complained of increasing dyspnoea and decreased exercise tolerance. Laboratory: NT-proBNP was 456 pg/mL. Echocardiography showed no dynamics. The results of subsequent RHC are shown in *Table 1*.

**Table 1:** Results of right heart catheterization in Patient 2

Measure	6 months after delivery (prior to/after the test with iloprost)		9 months after delivery, on PAH therapy
	Prior to the test	Iloprost 20 μg	
Pulmonary artery pressure, mm Hg	120/97/81	106/86/66	101/85/70
Aortic pressure, mmHg	122/91/69	115/83/60	100/86/79
Right atrium pressure, mm Hg	7	8	—
Pulmonary artery wedge pressure, mmHg	18	14	18
Pulmonary vascular resistance, Wood units	18.3	17.6	14.8
Cardiac index, L/min/m^2^	4.2	4	4.6
Mixed venous oxygen saturation, %	75.3/72.6		68.9

Nine months after delivery, the patient complained of increasing dyspnoea and decreased exercise tolerance. Laboratory: NT-proBNP was 456 pg/mL. Echocardiography showed no dynamics.

The results of subsequent RHC are shown in *Table 1*.

## Discussion

Descriptions of clinical cases of this defect in adults are very rare.^6,7^ Among the described cases, there are patients with and without Eisenmenger syndrome.^8,9^ The sources describe several clinical cases of patients with uncorrected aortopulmonary window defect, severe PAH, and completed pregnancy. Despite the fact that nowadays pregnancy in patients with PAH is considered safer, the mortality rate remains extremely high,^10,11^ reaching 12–20%. Labour and the early postpartum period should be attributed to the periods of greatest risk.^12,13^ Su-Mei et al.^14^ described a clinical case of low-symptomatic aortopulmonary window in a patient who successfully completed three pregnancies by vaginal delivery. There is no information on the course of previous pregnancies and deliveries in this clinical case. Thirunavukkarasu et al.^15^ described the cardiac autopsy findings of a woman with aortopulmonary window complicated by Eisenmenger syndrome. Death occurred at 21 weeks of gestation at the age of 20. Two clinical cases showing the development of Eisenmenger syndrome associated with aortopulmonary window defect with decompensation of heart failure (HF) in the postpartum period have also been described.^16,17^ However, the authors of these publications do not describe the course of pregnancy and delivery. In this paper, we present a novel detailed description of the course of pregnancy, delivery, and postpartum period in patients with aortopulmonary window defect, severe PAH, and Eisenmenger syndrome. Surgical repair at early age remains the main method of congenital heart disease treatment. Progression of the defect leads to irreversible changes that make surgical correction impossible.^4,18^

## Conclusion

Early termination of pregnancy is the most preferable option for patients with aortopulmonary window defect complicated by PAH and Eisenmenger syndrome. If the decision is made to preserve the pregnancy, these patients should be observed in specialized centres by a multidisciplinary team of healthcare professionals, due to the high risk of cardiovascular, obstetric complications, and death. Earlier verification of the diagnosis, active monitoring of the pregnancy, a carefully elaborated plan of therapies, and the timing and mode of delivery are the key aspects in the successful management of pregnant patients in this risk group.

## Limitations of approach in this clinical case

According to clinical guidelines, the examination as CT aorta or brain is contraindicated during pregnancy.^16^

## Lead author biography


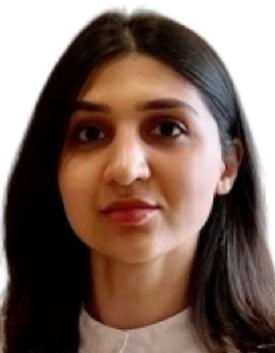
Sefiyeva G.G. is a cardiologist in Almazov National Medical Research Centre. Her main areas of scientific activity are congenital and acquired valvular heart disease and clinical features of the pregnancy in patients with prosthetic heart valves.

## Supplementary material

Supplementary material is available at *European Heart Journal – Case Reports* online.

## Supplementary Material

ytad501_Supplementary_Data

## Data Availability

The data underlying this article are available in the article and in its online supplementary material.
